# The Effect of Chamomile on Pain and Menstrual Bleeding in Primary Dysmenorrhea: A Systematic Review

**DOI:** 10.30476/ijcbnm.2021.87219.1417

**Published:** 2021-07

**Authors:** Azin Niazi, Maryam Moradi

**Affiliations:** 1 Department of Midwifery, School of Nursing and Midwifery, Mashhad University of Medical Sciences, Mashhad, Iran; 2 Nursing and Midwifery Care Research Center, Mashhad University of Medical Sciences, Mashhad, Iran

**Keywords:** Bleeding, Chamomile, Dysmenorrhea, Menstruation, Systematic Review

## Abstract

**Background:**

Primary dysmenorrhea is characterized by pain during menstruation without any pelvic pathology. It is a common problem among females in their reproductive age which is caused by increased production of prostaglandin in the endometrium as one of leading causes. Chamomile extract ceases the production of prostaglandins and leukotrienes. The aim of this study was to systematically review the clinical trials to determine the effect of Chamomile on pain and menstural bleeding in primary dysmenorrhea.

**Methods:**

Search process to find relevant articles was conducted on electronic Iranian (MagIran, SID) and international databases (Google Scholar, Science Direct, PubMed, ProQuest, Cochrane library, Scopus, Web of Science and EBSCO), using English keywords and Persian equivalents such a “Dysmenorrhea”, “Pain”, “Menstrual bleeding” and “Chamomil” without a time limit until March 2020. Irrelevant, duplicate, descriptive, or qualitative studies were excluded. To evaluate the quality of articles, we used the Cochran’s Risk of Bias tool.

**Results:**

Among124 articles found in the initial search, finally 7 clinical trials (with a sample size of 1033) were systematically examined. Two out of 7 studies examined the effect of Chamomile on the pain of primary dysmenorrhea, 2 studies on the effect of Chamomile on menstrual bleeding volume, and 3 on the effect of Chamomileon pain and menstural bleeding in primary dysmenorrhea.

**Conclusion:**

Based on results of the most reviewed studies, Chamomile can be considered as an effective treatment for primary dysmenorrhea and reducing menstrual bleeding.

## INTRODUCTION

Menstrual disorders include dysmenorrhea, increased or decreased duration, and volume of menstrual bleeding, which is very common among women. ^1 , 2^ Dysmenorrhea is a common gynecological disorder affecting about 50% of women of reproductive age. ^3^ The primary dysmenorrhea is menstrual pain without pelvic pathological causes which usually appears within 1-2 years after menarche at the same time as the ovulation cycles stabilize, and it may take 48-72 hours per cycle. ^4 , 5^ The prevalence of dysmenorrhea in Iran is reported to be about 95%, and about 1% of women can not perform their work for 1-3 days per month due to severe dysmenorrhea;1 therefore, dysmenorrhea is one of the main factors disrupting the quality of life and social activity. ^6^

Severe menstrual bleeding (menorrhagia) is another common gynecological problem, affecting about 30% of women of reproductive age. ^7^ Although it does not increase mortality in women, it has physical, psychological, and social consequences, such as iron deficiency anemia, reduced quality of life, and increased medical care cost. ^8^ According to the World Health Organization, 18 million women have menorrhalgia worldwide. ^7^

Increased production of prostaglandin in the endometrium is one of the accepted hypotheses for the mechanism of dysmenorrhea; therefore, prostaglandin inhibitors are the first line of treatment. ^9 , 10^ Side effects of medical treatment include gastrointestinal upset in the form of nausea and vomiting, kidney disorders, stomach ulcers, dizziness, tinnitus, allergic reactions, blood and liver side effects, bleeding, and spotting symptoms. ^11^

Nowadays, women around the world have turned to medical herbs to address reproductive problems such as menstruation, infertility, pregnancy, and childbirth. ^12^ Herbal products are one of the most basic methods of combating diseases, which have a significant advantage over chemical medicines due to more acceptability and fewer side effects. ^13 , 14^ Thyme, Fennel, Calendula, Dill extract, Saffron, Teucriumpolium, Bromelain, Fenugreek, Rosemary, and Yarrow have been reported to be effective on dysmenorrhea and menstrual disorders. ^15^ Due to inadequate number of studies and poor methodology, it is not possible to draw definite conclusions about the effect of these plants. ^15 - 19^

Chamomile, with the scientific name of Matricariachamomilla, is one of the most widely used medicinal plants. ^20^ According to studies on Chamomile so far, its effects on stomach pain, irritable bowel syndrome, insomnia, and wound healing have been confirmed. ^21^ Pure Azoline is one of the effective compounds in this plant that has anti-inflammatory and antiseptic effects. Apigenin and methoxy-coumarin have antispasmodic properties. ^22 , 23^ The anti-inflammatory effects of Chamomile are mostly due to compounds such as Matrisin and Bisabolol. There is also evidence of flavonoids with similar functions to benzodiazepines and phytoestrogens in Chamomile, which has positive sedative effects. ^24 , 25^ A study in Iran showed that edible Chamomile capsules had a significant effect on reducing primary dysmenorrhea. ^26^ However, in another study on comparing the effect of Chamomile and Yarrow capsules on the severity of primary dysmenorrhea, both Yarrow and Chamomile capsules reduced the severity of the pain. ^27^

Nevertheless, Yarrow capsules were more effective in reducing the severity of menstrual pain due to their long-lasting sedative properties. ^27^ In a study with the aim of comparing Chamomile and mefenamic acid capsules in hemorrhage of menstruation, Chamomile consumption was effective in reducing menstrual bleeding. ^28^ In another study, the rate of menstrual bleeding in the Chamomile group decreased compared to the placebo group. However, this difference between the two groups was not significant. ^29^ The results of a systematic review (2019) investigated three studies ^26 , 30 , 31^ on the field of the effect of Chamomile on the primary dysmenorrhea along with other medicinal-chemical plants, acupuncture, and acupressure. The results showed the effectiveness of complementary medicine on primary dysmenorrhea. However, further studies with a more robust methodology are recommended. ^32^ A review study examined 16 types of medicinal plants, most of which had a positive effect on the primary dysmenorrhea. In these reviews, there was a study on the effect of Chamomile on primary dysmenorrhea. ^19 , 26^ In another review study, the effect of Chamomile on dysmenorrhea was evaluated in three studies. ^30 , 32^ Although chamomilla has proved effective, further clinical trials are necessary with the same scale for measuring pain, investigating possible side effects, observing blinding rules and randomization so as to provide a definitive conclusion about their effective use and dose. ^33 , 34^ Regarding the side effects, drowsiness was the only reported complication after the oral consumption of Chamomile. ^28^

In order to access the effective and safe treatments, several studies conducted so far aimed at determining the effect of Chamomile on dysmenorrhea and the volume of menstrual bleeding; however, the previous review failed to provide definite conclusion on this field, specially due to small number and poor methodology of articles. ^19 , 32 , 34^ Given that a systematic review attempts to collate all empirical evidence that fits pre-specified eligibility criteria in order to answer a specific research question, ^35^ and lack of definitive conclusions about the effect of Chamomile, the aim of this review was to systematically summarize and critically evaluate the effect of Chamomile on pain and menstrual bleeding in primary dysmenorrhea.

## MATERIALS AND METHODS

In this systematic review, the research question was determined based on population, intervention, comparison and outcomes (PICO). Then, the search process to find relevant articles was conducted on electronic databases including in Iranian (MagIran, SID) and international databases (Google Scholar, Science Direct, PubMed, ProQuest, Cochrane library, Scopus, Web of Science and EBSCO). To search for articles, we used English keywords and Persian equivalents including: “Dysmenorrhea”, “Menstruation”, “Menorrhagia”, “Chamomile”, “Herbal Medicines”, “Menstrual Bleeding”, “Aromatherapy”, “Menstrual Pain”, “Period Pain”, “Pain”, “Pain Relief” and all possible combinations of these words with the OR, AND Boolean operators without time limit until March 2020.

**Figure 1:**
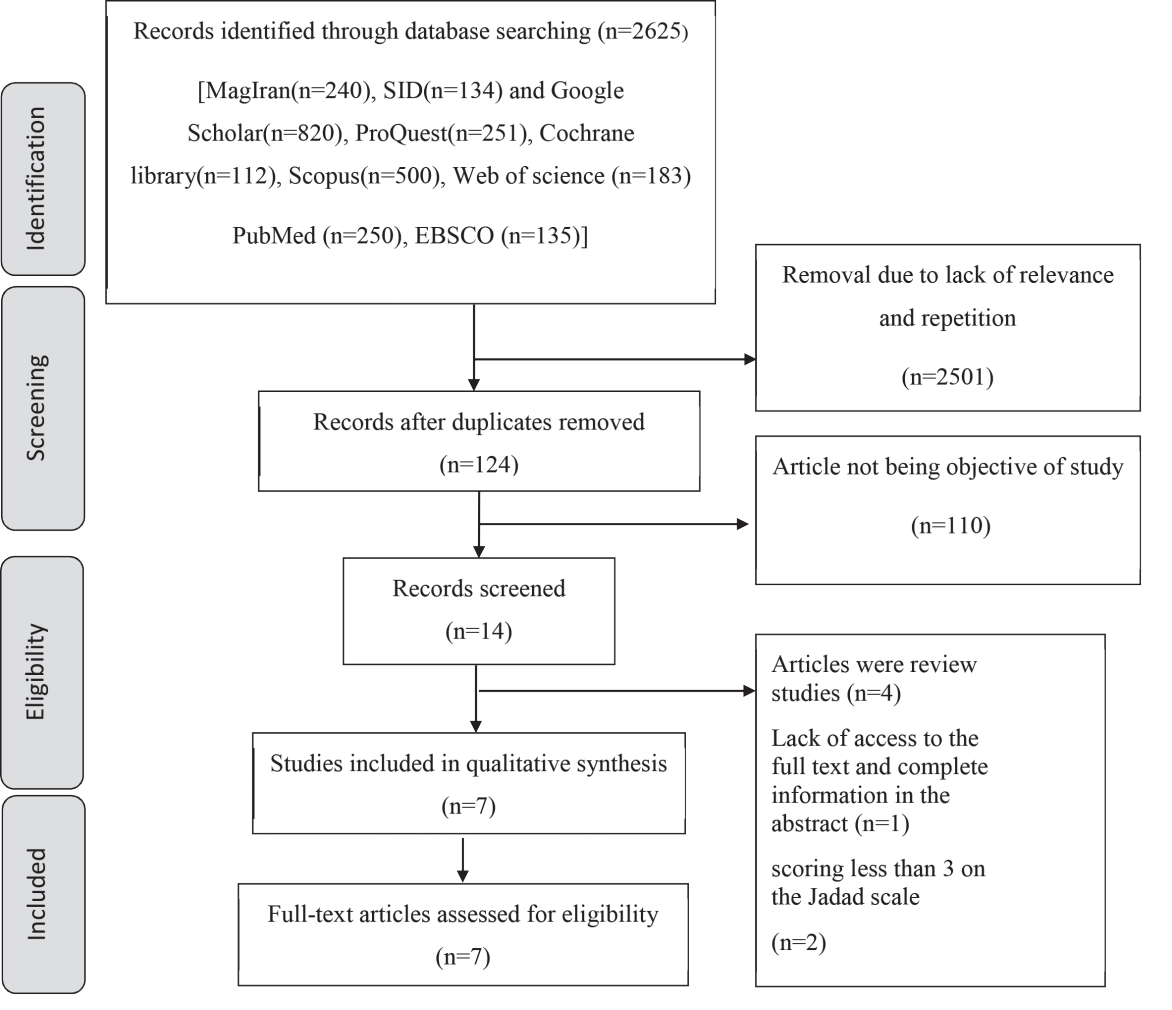
Study selection procedure (PRISMA flowchart).

The population studied were women of reproductive age with primary dysmenorrhea. They used Chamomile to relieve menstrual cramps or reduce bleeding and alternative intervention or control performed in the other group. The main criteria for the inclusion of articles into this structured review were randomized human clinical trials published in Persian and English, which examined the effect of Chamomile on primary dysmenorrhea and menstrual bleeding, with standard tools to assess pain and bleeding. Exclusion criteria included scores lower than 3 on the scale Jadad, irrelevant and duplicate studies, lack of study goal achievement, not an original study, pilot study and descriptive or qualitative studies. Methods of presenting materials, including analysis and interpretation, determining the problem under study, and collecting the findings were based on the Preferred Reporting Items for Systematic Reviews and Meta-Analyses (PRISMA) Systematic Studies Reporting System. ^36^ In order to select articles and additional data, first, all the articles used the desired keywords in the title or abstract sections included in the study. Quality review and extraction of articles were conducted by experts in the field of review who have a history of systematic performance. A preliminary review of the article was conducted on the abstract and irrelevant and repetitive items were removed; then, the full text of the articles was reviewed. A flowchart (Figure 1 ) shows the process of selecting articles and the reasons for the withdrawal of the article from the study.

In order to maximize the comprehensiveness of the search, the list of sources of related articles was reviewed manually. Jadad scale was used to evaluate the articles. This scale examined the articles based on the clinical trials of the study, the method of randomization, blindness, and the way the study was performed and followed by patients. The minimum score in this scale was 1, and the maximum was 5. In case of disagreement between the two researchers, a third person was used to approve or reject the article. According to the Jadad Scale, articles that scored 3 or more were included in the study. ^37^ In order to evaluate the quality of the articles, included studies were investigated in terms of selection bias (production of random sequencing and allocation concealment), execution (blinding participants and evaluators), diagnosis (blinding statistical analyst), sample shedding (exclusion after randomization study) and reporting (selective report of consequences). For this purpose, the Risk of Bias instrument of the Cochran group was used. ^38^

## RESULTS

Among the 124 articles found in the initial search, 14 articles were reviewed after removing duplicate and unrelated items. four studies were excluded because they were review studies ^19 , 32 , 34 , 39^ and 1 study was excluded due to lack of access to the full text and lack of provision of the complete information in the abstract. The full text of the remaining 9 studies were reviewed and 2 studies were excluded due to obtaining score 3 in the Jadad Scale. ^30 , 31^ Finally, 7 clinical trials (with a sample size of 1033 people) were systematically examined (Figure 1 ). Examples of search process in an electronic database to find relevant articles are showed in Appendix 1.

Two out of 7 studies examined the effect of Chamomile on pain in primary dysmenorrhea, 2 were conducted on the effect of Chamomile on menstrual bleeding volume, and three studies examined the effect of Chamomileon pain and menstrual bleeding in primary dysmenorrhea. A summary of the studies reviewed is shown in Table 1

**Table1:** Characteristics of the studies included in the systematic review

Author/year/reference number	Setting	Method	Objective	Tool	Research sample	Intervention group	Control group	Results	Side effect	Jadad scale score
Karimian et al., 2013^40^	Iran	Clinical trial	Comparing the effect of Chamomile and Mefenamic capsules on primary dysmenorrhea	McGill Pain Questionnaire Higham Questionnaire	N=90 Female students with primary dysmenorrhea	250 mg of Chamomile every 8 hours for 48 hours before menstruation until 24 hours after that with a duration of 2 cycles. 45 subjects	250 mg of Mefenamic Acid capsules every 8 hours from 48 hours before menstruation until 24 hours after that with a duration of 2 cycles. 45 subjects	Chamomile capsule is effective in reducing the intensity of primary dysmenorrhea and menstruation bleeding (P<0.005)	Drowsiness in Chamomile group (4 subjects) Constipation in Mefenamic Acid	5
Radfar et al., 2018^27^	Iran	Clinical trial	Comparing the effect of Yarrow and Chamomile on menstrual pain	VAS^a^ score	N=50 Female students with primary dysmenorrhea	First group: 250 mg Chamomile capsule every 8 hours 24 subjects During the first 3 days of menstruation for a duration of 2 cycles	Second group: 250 mg Yarrow capsule every 8 hours 26 subjects During the first 3 days of menstruation for a duration of 2 cycles	Both Chamomile and Yarrow capsules reduced the pain intensity. But Yarrow capsule with its long term sedative property was more effective in reducing menstrual pain	No reference was made	4
Modarres et al., 2011^26^	Iran	Clinical trial	Comparing the effect of Chamomile and Mefenamic capsules on initial dysmenorrhea	VAS score Menstrual Bleeding Checklist	N=160 Female students with primary dysmenorrhea	400 mg Chamomile every 6 hours during the first 3 days of menstruation for the duration of 2 cycles 80 subjects	250 mg Mefenamic Acid every 6 hours during the first 3 days of menstruation for a duration of 2 cycles 80 subjects	Chamomile capsule is effective in reducing the intensity of initial dysmenorrhea (P< 0.001) Both Mefenamic Acid and Chamomile reduced the menstruation bleeding after 2 cycles	No reference	was made	5
Samadi et al., 2015^43^	Iran	Semi-experimental	Investigating the effect of consumption of a compound of Fennel, Chamomile, Ginger in reducing the pain intensity of initial dysmenorrhea	VAS score	N=90 Female students with primary dysmenorrhea	A compound tea comprised of Fennel, Chamomile, and Ginger in 300cc of boiling water, 2 cups daily from a week before menstruation until the fifth day of menstruation for 12 days with a during 2 cycles (a total of 24 days, 2 cups each day) (90 subjects)	No control group	Consumption of a compound of Fennel, Chamomile, and Ginger was effective in relieving dysmenorrhea symptoms	No reference was made	4
NajafiMollabashi et al., 2020^29^	Iran	Clinical trial	Investigating the effect of Chamomile on duration, amount, and distance between menstruation bleeding	Higham Charts	N=118 Female students with primary dysmenorrhea	250 mg of Chamomile powder third times a day 7 days before menstruation until the onset of menstruation during a cycle 59 subjects	Placebo 59 subjects	Chamomile reduces menstruation bleeding	No reference was made	4
Ehsani et al., 2013^41^	Iran	Clinical trial	Investigation of the effect of herbal plants (Thyme, Salvia, Chamomile) on the menstruation bleeding intensity	Researcher made Questionnaire	N=125 Female students with primary dysmenorrhea	Brewed herbal plants (Thyme, Salvia, Chamomile) third times a day during the first 3 days of menstruation for during of 3 cycles 50 subjects	Control group 1: Placebo 45 subjects Control group 2: MefenamicAcid 30 subjects	Consumption of these brewed plants reduced menstruation bleeding intensity (p=0.000)	No complication	5
Shabani et al.(2020)^42^	Iran	Clinical trial	Chamomile, Ginger, Mefenamic acid, Chamomile-Ginger on the intensity of menstrual bleeding and dysmenorrhea	Pictorial Blood Loss Assessment Chart (PBACs) VAS score	N=400 Female students with primary dysmenorrhea	group 1; 1000 mg of ginger root powder plus honey; group 2: 500 mg of Chamomile with honey; group 3: 1000 mg of ginger and 500 mg of Chamomile three times daily form 2 days before menstruation to the first 3 days for during of 2 cycles	Mefenamic acid (250 mg)	Mefenamic acid also had a better effect on reducing bleeding than other interventions (P=0.008). The severity of pain, and bleeding rate were significantly decreased in all 4 groups (P=0.001).	Most of the complications were related to the ginger group)hot flash Allergy( gastrointestinal	5

^a^Visual analog scale

The largest and smallest samples had 400 and 50 patients, respectively. Five controls group were given placebo capsules and mefenamic acid, ^26 , 29 , 40 - 42^ one control group recieved Yarrow capsule, ^27^ and one study lacked a control group, ^43^ while individuals in the treatment group were given oral capsules Chamomile, ^26 , 27 , 29 , 40^ (Thyme, Salvia, Chamomile), ^41^ Chamomile with Honey, ^42^ and Chamomile in combination with Ginger and Fennel. ^42^ The daily dose of Chamomile ranged from 250 mg to 500 mg. The most common duration of Chamomile treatment was two menstruation cycles.

The number of days of treatment was not the same among the studies. Karimian et al. (2013) tested a three-day regimen (48 hours before menstruation until 24 hours after that). ^40^ Radfar et al. (2018), Modarres et al. (2011) and Ehsani et al. (2013) tested a three-day regimen (first 3 days of menstruation). ^26 , 27 , 41^ In the study carried out by Samadi et al. (2015), the participants were given combination of Fennel, Chamomile and Ginger and tested a twelve-day regimen (one week before menstruation until the fifth day of menstruation for two cycles). ^43^ Najafi Mollabashi et al. (2020) tested a seven-day regimen for one cycle ^29^ and in a study by Shabani et al. (2020), treatment was taken two consecutive cycles, three times daily from 2 days before menstruation to the first 3 days. ^42^

To measure the pain severity, four studies used the visual analogue scale (VAS). ^26 , 27 , 42 , 43^ One study measured pain severity using the McGill Pain Questionnaire. ^40^ Another study measured bleeding ScalePictorial Blood Loss Assessment Chart (PBACs). ^42^ In two trials, bleeding was measured through the Higham Questionnaire. ^29 , 40^ One study measured bleeding using the Menstrual Bleeding Checklist Researcher ^26^ and another one used a researcher made Questionnaire. ^41^

Two studies examined the effect of Chamomile on pain in primary dysmenorrhea which showed the effectiveness of Chamomile on primary dysmenorrhea, two studies examined the effect of Chamomile on menstrual bleeding volume which showed the effectiveness of Chamomileon menstrual bleeding volume, and three studies were done on the effect of Chamomile on pain and menstrual bleeding in primary dysmenorrhea, which showed the effectiveness of Chamomile on dysmenorrhea and menstrual bleeding.

Karimian et al. (2013) showed that both Chamomile and mefenamic acid could reduce the severity of pain and hemorrhage (P<0.05). ^40^ Radfar et al. (2018) showed a statistically significant difference between the two groups of Yarrow and Chamomileon menstrual pain in the first cycle (P<0.05). However, this difference between the two groups was not significant in the second cycle (P>0.05). ^27^ In a study by Modarres et al. (2011), the mean of pain intensity was significantly different between the two groups (P<0.001). The mean bleeding amount decreased after the two treatment cycles in both groups, but there was no statistically significant difference between the two groups (P>0.05). ^26^ In a study by Samadi et al. (2015), consumption of a compound of Fennel, Chamomile, and Ginger was effective in relieving dysmenorrhea symptoms. ^43^ In a study by Najafi Mollabashi et al. (2020), the mean of bleeding in the Chamomile group reduced before and after the intervention (P<0.001). There was no statistically significant difference between the duration of menstruation and the intervals between the periods between and within groups. ^29^ In a study by Ehsani et al. (2013), the results showed that there was a statistically significant difference in the number of bleeding days and the number of pads used in the intervention group (Thyme, Salvia, Chamomile) before and after treatment (P=0.0001). ^41^ In a study by Shabani et al. (2020), the results showed that the severity of pain and amount of bleeding were significantly decreased in all 4 groups (Ginger, Mefenamic acid, Chamomile-Ginger) (P=0.001). ^42^

**Figure 2:**
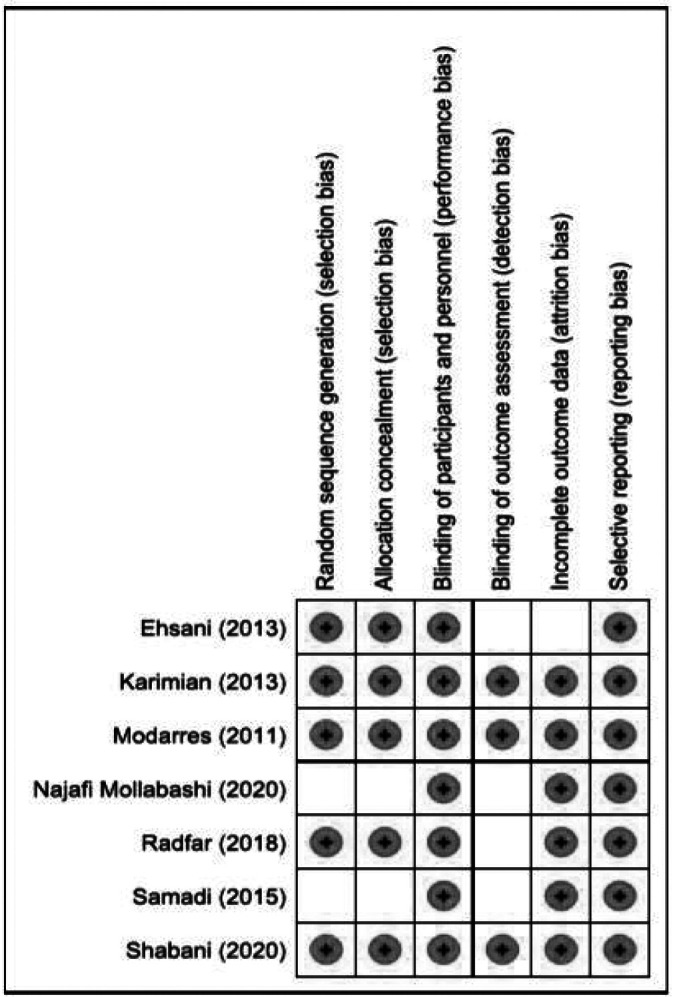
Risk of bias summary; review of authors’ judgements about each risk of bias item for each included study.

**Figure 3:**
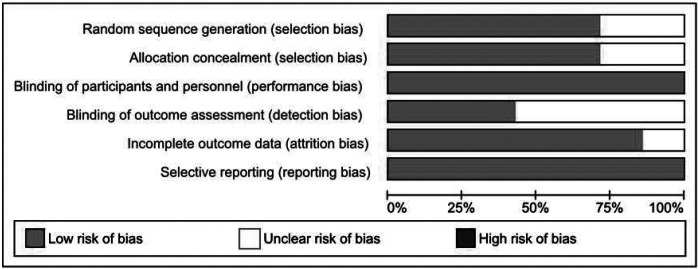
Risk of bias graph; review of authors’ judgements about each risk of bias item presented in percentages across all studies.

The quality of the articles included was systematically reviewed using Cochran’s Risk of Bias tool. In terms of random sequence Bias, the two studies were in a vague range due to the lack of explanation of how randomization occurred. ^29 , 43^ Low Bias was determined in two studies due to the use of random sequence production software ^43 , 44^ and in three studies due to the use of cards to assign the subjects into control and intervention groups, ^26 , 27 , 41^ respectively. In terms of allocation concealment Bias, two studies had low Bias due to the use of computer software ^40 , 42^ and the three other studies had low Bias due to the use of cards. ^26 , 27 , 42^ Two studies were in a vague range due to lack of sufficient information to judge. ^29 , 42^ In terms of implementation Bias, five studies were performed by the two-sided blinding ^27 , 29 , 42 , 43^ and two studies were performed by three-sided blinding. ^26 , 40^ These studies showed to have a low Bias in terms of implementation Bias. In terms of diagnostic Bias, in five studies, the information needed to determine awareness of the data analyst from the assignment of individuals in treatment or control groups was not available. ^27 , 29 , 42 , 43^ Therefore, these five studies were in a vague range in the evaluation. In terms of sampling Bias, the participants of six studies attended the study from randomization to the analysis of the results. ^26 , 27 , 29 , 40 , 42 , 43^ Thus, these studies had a lowbias in terms of sampling shedding bias. In terms of the reporting bias, all seven published articles contained all the expected consequences. According to the evaluation of the quality of studies entered into this systematic review using the Risk of bias tool of Cochran Group, most studies had a proper methodology. In sum, the risk of bias for each study is presented in Figures 2 and  3.

## DISCUSSION

This was a systematic review to determine the effect of Chamomile on pain and menstural bleeding in primary dysmenorrhea. This review summarizes evidence from seven studies. The results of the studies suggest a pattern of oral Chamomile as a potentially effective treatment for pain in dysmenorrhea. Overall, Chamomile was reported as more effective for pain relief than placebo, and NSAIDs. The etiology of primary multifactorial dysmenorrhea, including the synthesis and secretion of prostaglandin F2α, increases in vasopressin and oxytocin is followed by an increase in prostaglandin secretion. ^44^ Chamomile extract interrupts cyclooxygenase and lipoxygenase; thus, stopping the production of prostaglandins and leukotrienes. ^21 , 45^ The anti-inflammatory and analgesic effects of Chamomile have been attributed to the compounds such as Matrisin and Bisabolol, and their oxides. ^46 - 48^ In one study, Chamomile-like Mefenamic Acid was effective and pain in the second cycle was more pronounced than in the first cycle. ^40^ In another study, Chamomile capsule was effective in reducing the severity of pain after two treatment cycles in patients with primary dysmenorrhea, ^26^ which may indicate that treatment for several cycles is needed to achieve the acceptable effect of the medicine. The results of a study showed Chamomile, in combination with Fennel and Ginger, had a powerful impact on reducing the severity of dysmenorrhea. ^43^ As a combined treatment has been used in this study, distinguishing the positive effect regarding the of Chamomile is ambiguous; whether Chamomile has reduced severity of dysmenorrhea or the other two plants or positive results has been caused by combined effect of the three plants. In another study, Chamomile and Yarrow capsules were effective in reducing the severity of primary dysmenorrhea. However, the severity of pain in the Yarrow capsule group was significantly higher. ^27^ Differences in dosege and time of treatment use in studies could be one of the factors influencing the effect of Chamomile on the severity of menstrual bleeding.

This review summarized evidence from five studie on the efficacy of Chamomile on menstrual bleeding for primary dysmenorrhea. All five studies that examined the effect of Chamomile on menstrual bleeding emphasized its effectiveness. One of the most probable causes of heavy menstrual bleeding is an increase in the production of uterine prostaglandins. Given that E2 and F2α prostaglandins increase the blood flow to the uterus and E2 prostaglandin is a vasodilator, the likelihood of menstrual bleeding increases. Therefore, inhibition of prostaglandin synthesis can improve menstrual bleeding in women. ^44 , 49 , 50^ In the study, the effect of a combination of three herbs, including Chamomile, Salvia, and Thyme, on the severity of menstrual bleeding was examined. The effectiveness of these three plants has been proven. The combined extract reduced the number of bleeding days, number of pads used, and excretion of clots. ^42^ Nevertheless, the combination of Ginger and Chamomile did not cause synergy of the effects, and this combination was not significantly superior to Ginger, Chamomile, or honey for the treatment of menstrual bleeding. Therefore, the consumption of Ginger or Chamomile each had better effects in isolation. ^42^ Thus, this effect should always be compared to each of its effective components in order to avoid imposing the undesirable side effects of the medicine on the patient. In another study, Chamomile was effective in reducing menstrual bleeding. Chamomile has anti-prostaglandin properties. By interrupting the cyclooxygenase chain, it limits the production of prostaglandins and leukotrienes. ^29^ In a study, 85% of the Mefenamic Acid group members had moderate bleeding after two cycles of treatment. This rate was 96.3% in the Chamomile group, and two cycles of Chamomile treatment reduced the menstrual bleeding. ^26^ In two other studies, Chamomile was effective in reducing the menstrual bleeding. ^27 , 41^

One of the strengths of the present study was evaluation of the quality of the articles entered using the Jaddah scale and Risk of bias of the Cochran group.

Limitations of this study included the lack of meta-analysis which was due to the difference in the amount of extract used, the method of using Chamomile, time of the intervention for reaching a definite conclusion about the amount and time of Chamomile usage for primary dysmenorrhea and menstrual bleeding.

## CONCLUSION

According to the results of the majority of studies reviewed, Chamomile can be considered as an effective treatment for primary dysmenorrhea and reduction of menstrual bleeding. Further studies are suggested to provide more robust scientific evidence on the most effective dose, their possible effects and side effects, and the possibility of meta-analysis. Considering the rising popularity of medical herbs in women around the world, healthcare providers including midwives could consider evidence-based effective herbal treatments like Chamomile to manage pain and menstrual bleeding in primary dysmenorrhea. 
